# Revisiting the Baltimore–Bullock's Oriole hybrid zone reveals changing plumage colour in Bullock's Orioles

**DOI:** 10.1098/rsos.221211

**Published:** 2022-12-14

**Authors:** Young Ha Suh, Russell A. Ligon, Vanya G. Rohwer

**Affiliations:** ^1^ Department of Ecology and Evolutionary Biology, Cornell University Museum of Vertebrates, Ithaca, NY 14853, USA; ^2^ Cornell Lab of Ornithology, Ithaca, NY 14850, USA

**Keywords:** Baltimore–Bullock's hybrid zone, oriole, change in colour, carotenoid, introgression

## Abstract

Hybrid zones are dynamic areas where populations of two or more interbreeding species may change through an influx of novel genetic material resulting from hybridization or selection on standing genetic variation. Documenting changes in populations through time, however, is challenging because repeated samples are often missing or because long-term storage can affect trait morphologies, especially colour traits that may fade through time. We document a change in carotenoid-based orange breast feathers of Bullock's Orioles (*Icterus bullockii)* from the Great Plains hybrid zone, USA. Contemporary Bullock's Orioles are more orange than historic individuals from the same location sampled approximately 60 years ago. Spectrophotometry revealed that contemporary Bullock's Orioles resemble orange colour profiles of Baltimore Orioles (*I. galbula*), the species with which they hybridize. Fading or changes in diet hypotheses do not appear to explain the shift in colour we report for Bullock's Orioles. We propose that these changes in colour are facilitated through introgression with Baltimore Orioles, and favoured by females that choose brighter, more orange males. Our study highlights the long memory of natural history collections and how they offer new insights to the dynamic roll of hybrid zones in trait evolution between interacting species.

## 1. Introduction

Hybrid zones have the potential to promote rapid trait evolution within species through several different mechanisms. Social interactions between species can drive character evolution such that traits diverge (e.g. character displacement [1]) or converge (e.g. mimicry [2–4]) in areas of sympatry. Traits important to species recognition and signalling to conspecifics should evolve rapidly in hybrid zones and in areas of sympatry, especially if the costs of hybridization or the costs of social interactions for shared resources are high [1,5,6]. Hybridization can also introduce novel genetic material for selection to act upon, pushing trait evolution in new directions or to higher optimums that were otherwise limited by genetic variability within species [7–9]. Finally, hybrid zones often rest at the limits of species ranges, and these environments may expose populations to different selective regimes, driving trait evolution in different directions compared to populations at the centre of the range [10]. Understanding which traits are changing, as well as the pace and direction of these changes, can help understand which mechanisms are responsible for trait evolution in hybrid zones.

Documenting changes in traits between interacting species in hybrid zones can be challenging. Historical samples from focal populations may provide a baseline for assessing change through time, but such a baseline is often missing or inferred from populations beyond the study site, potentially confounding geography with shifts in phenotype. Even in populations for which historical samples from the same location exist, some traits may appear to change through time because they fade in colour or alter their shape or size with prolonged storage [11,12]. Finally, the unpredictability of knowing which traits to measure during the initial sampling effort further complicates tracking and understanding changes in ecologically and evolutionary important traits through time. Collectively, these challenges highlight some of the reasons why natural history collections play a crucial role in documenting changes in populations through time [13,14].

**Figure 1.**
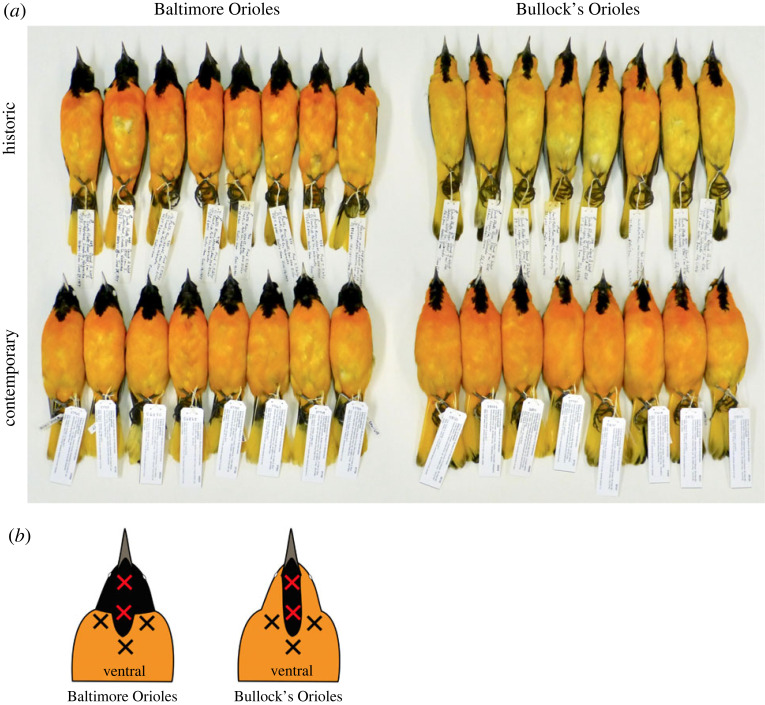
(*a*) Baltimore Orioles on the left show no noticeable shift in colour between historic and contemporary specimens, whereas Bullock's Orioles on the right show a noticeable change in orange colour over the same time period of approximately 60 years. (*b*) Illustration of the five measurements taken from the breast and throat of each species.

Here, we describe a change in orange plumage colour in adult male Bullock's Orioles (*Icterus bullockii*), using specimens that show little to no signs of hybridization from the Great Plains hybrid zone [15–17]. The change in colour we describe has occurred over approximately 60 years/generations. The breast and bellies of adult male Bullock's Orioles are typically a yellow-orange compared to the orange breast and bellies of adult male Baltimore Orioles (*I. galbula*) (figure 1). The change in orange colour we document is pronounced in Bullock's Orioles from within the hybrid zone but absent in individuals from outside the zone (see analyses below), suggesting that this change is geographically restricted. Moreover, this change in colour resulted in Bullock's Orioles matching orange colour profiles of Baltimore Orioles, the species with which they hybridize.

Several different hypotheses could explain the change in orange colour characters we document in orioles. First, fading of older specimens could explain why contemporary specimens appear more orange than historical ones [11], and this hypothesis is especially relevant to the carotenoid-based pigments in the orange breast and belly feathers of orioles, as they are more prone to fading than melanin-based pigments [18]. Second, changes in land use in the southwestern United States and northwestern Mexico [19], where Bullock's Orioles replace (i.e. moult) their feathers [20,21], could have changed the availability of carotenoid rich food resources, potentially driving these changes in colour [22–24]. Third, adaptive introgression from the brighter Baltimore Orioles into Bullock's Orioles may affect the conversion or expression of carotenoid-based pigments, allowing Bullock's Orioles that now possess these genes to produce colours similar to those of Baltimore Orioles [25]. Fourth, selection acting on standing genetic variation for more orange male Bullock's Orioles may have resulted in the evolution of these plumage characters. For the third and fourth hypotheses, we can envision at least two forms of social selection—sexual selection driven by females [26] or competition with Baltimore Orioles (or other species within the hybrid zone) [5]—that could be responsible for favouring more orange plumages in adult male Bullock's Orioles.

We leverage a unique dataset that includes repeated sampling of the same sites within the well-studied Baltimore–Bullock's Oriole hybrid zone in Nebraska and Colorado, to better understand changes in the orange breast and belly feathers observed in adult male Bullock's Orioles. Below, we outline predictions and tests for two of the four possible hypotheses—fading and changes in diet—that could be responsible for these changes in colour.

### 1.1. Fading hypothesis

Museum specimens can fade over time [11,12]. If fading is responsible for the change in orange colour in Bullock's Orioles, then we should see this same pattern in our sample of Baltimore Orioles: duller historic specimens and brighter contemporary ones. Historic specimens of both oriole species have been prepared and stored in identical conditions for the same amount of time. Baltimore and Bullock's Orioles also show similar carotenoid profiles in their feather chemistry [18,27], suggesting both species should be similar in their susceptibility to fading. Moreover, because contemporary specimens were collected as part of a larger effort examining hybrid zone dynamics through time, historic and contemporary specimens of both species are paired by location. Thus, if fading explains the change in colour we report for Bullock's Orioles, we should see similar changes in orange colour profiles in Baltimore Orioles: duller historic specimens and brighter contemporary ones.

### 1.2. Changes in diet hypothesis

Birds cannot generate carotenoid colours on their own and must acquire these pigments through their diet [22,23]. Therefore, the changes in orange carotenoid-based plumage of Bullock's Orioles could be explained by changes in land use and food resources on their moulting grounds [21]. To test this hypothesis, we compared colour profiles from a sample of migratory Bullock's Oriole specimens collected from outside of the hybrid zone but during the breeding season, which encompass (and exceed) the date range in our hybrid zone samples. The moult biology of Bullock's Oriole makes them well suited to evaluate this hypothesis. Bullock's Orioles depart their northern breeding grounds then migrate to the monsoon region of the American southwest and northwestern Mexico to replace their feathers [19]. Because Bullock's Orioles from across the breeding range moult in a common location, the shift in colour we observed in orioles collected from the hybrid zone should also be seen in those birds collected from outside of the hybrid zone if changes in land use or diet on the moulting grounds are responsible for this pattern. Rains in the monsoon region are patchy and unpredictable, suggesting that moult migrants, like Bullock's Orioles, should cover broad areas in search of habitat that has greened in response to rain, rather than being philopatric to specific moulting sites or habitat types [28]. Tracking data from Bullock's Orioles tagged at the same breeding site in southern British Columbia corroborate this suggestion [21], as individuals travelled to different locations within the monsoon region, suggesting that breeding sites do not predict molting sites. Thus, if changes in land use on the moulting grounds of Bullock's Orioles are responsible for the changes in colour in individuals from the hybrid zone, we should see parallel changes in colour in Bullock's Orioles collected from outside of the hybrid zone.

## 2. Methods

### 2.1. Choice of hybrid zone specimens and moult biology of orioles

In the 1950s, Sibley & Short [29] thoroughly sampled the Baltimore–Bullock's hybrid zone along the Platte River transect through Nebraska and Colorado. Recently (2016–2018), the Cornell University Museum of Vertebrates has resurveyed Sibley & Short's [29] Platte River transect of orioles and other avian hybrid zones, as part of a larger effort to examine hybrid zone dynamics through time [17,30]. These two sampling events were paired by location, and Baltimore and Bullock's Orioles were prepared using identical methods during each sampling event. Along the Platte River transect, the oriole hybrid zone rests near the Nebraska–Colorado border, with the highest frequency of phenotypic hybrids found between Big Springs, NE and Crook, CO. The most striking plumage characters indicative of hybrid ancestry are intermediate, orange and black facial feathers. Bullock's Orioles have mostly orange heads with a black eye-stipe, throat patch, and cap whereas Baltimore Orioles have completely black heads. Facial patterns of hybrids often include variable amounts of orange and black on the face, forehead, throat and ear feathers, but also intermediate amounts of white on the greater and lesser wing coverts, and orange and black on the tails (figure 1*a*; see Walsh *et al*. [17] for visuals of hybrid orioles). For our measures of plumage colour, we used 20 adult males from each species (with one small exception where we used 19 contemporary Baltimore Orioles) that showed little to no phenotypic signs of hybridization from each sampling period (i.e. 20 adult male historic Bullock's and 20 adult male contemporary Bullock's; same for Baltimore Orioles, with the exception noted above, totaling 79 individuals from the hybrid zone). While some individuals we used in colour measures showed subtle signs of hybridization, this did not influence measures of orange colour (see electronic supplementary material, figures S4 and S5).

Adult Baltimore and Bullock's Orioles differ in the timing, extent, and number of moults annually. Both species undergo a complete post-breeding moult (the prebasic moult), replacing all body and flight feathers. However, Baltimore Orioles undergo the prebasic moult on the breeding range prior to their autumn migration, whereas Bullock's Orioles first depart their northern breeding grounds and migrate to the American southwest and northwestern Mexico to undergo their prebasic moult, then continue their autumn migration south. This moult-related movement of Bullock's Orioles is observed among many Neotropical migrants that breed in western North America and is thought to have evolved in response to the late summer drought and insufficient food resources to support the prebasic moult on the breeding range [19]. In the spring, male Baltimore Orioles undergo some amount of prealternate moult, replacing some head, throat, body and flight feathers, whereas male Bullock's Orioles undergo little to no prealternate moult [31]. Regardless of differences in moult biology between oriole species, no orioles used in colour measures were actively moulting. Moreover, upon examining throat and chest feathers of both Baltimore and Bullock's Orioles used in colour analyses, we noticed no obvious differences in feather ages (e.g. slight fading or ragged, worn feather tips in some feathers but not others within an individual), suggesting that variation in the extent of the prealternate moult had minimal impact on our colour measures.

### 2.2. Measurement and analysis of reflectance spectra

We measured reflectance spectra from museum specimens of adult male Bullock's and Baltimore Orioles. We used the Ocean View spectrometer USB 2000+ (Ocean Optics; Dunedin, FL, USA) with a PX-2 light source and reflection probe to collect reflectance values for wavelengths between 300 and 700 nm (integration time, 100 ms; 10 readings averaged per recording; boxcar width 10). For each specimen, we took colour measures from three places on the orange breast and belly and two places on the black throat as a control for possible fading (figure 1*b*). We measured specimens by placing the probe at a 90° angle from each plumage surface. We measured each location 3 times (resulting in 15 measures per specimen) and used the average of ‘orange’ and ‘black’ measurements from an individual for further analyses. We reset the white standard between specimens. While measuring a subset of Bullock's Oriole specimens from outside the hybrid zone, there was an unknown interference that added two peaks in the spectra (see electronic supplementary material, figure S1). To account for this, we removed the peaks and extrapolated the spectra to smooth the curve, providing a more accurate measure of colour. This interference did not occur in measures of historic or contemporary Baltimore and Bullock's Orioles from the hybrid zone, and occurred in only 20 of 40 Bullock's Orioles from outside of the hybrid zone.

### 2.3. Avian colour models

Regardless of the mechanism(s) causing the change in orange colour observed in Bullock's Orioles, these changes must be perceived by birds to impact ecological and evolutionary dynamics of orioles in the hybrid zone. Models of avian vision show that many, if not most, species of birds have tetrachromatic vision that encompasses UV wavelengths and see a broader spectrum of light than humans [32–34]. While wavelengths of carotenoid colours are not within the UV spectrum, avian colour models should corroborate the shift in orange colour we noticed in Bullock's Orioles and potentially subtle differences in colour that may be perceived by birds.

To test how birds perceive colour differences, we used avian visual models to compare the perceptual distance between colours based on receptor-noise limited models [35,36] using methods from Maia & White [37]. We calculated colour distances using relative receptor densities {U, S, M, L} = {1, 2, 2, 4} and Weber fraction for L = 0.1. We used the ‘vismodel’ function in the pavo package that accounts for avian visual sensitivities while calculating quantum catches of photoreceptors then calculated the colour distances using the ‘coldist’ function [38].

We then compared (i) changes in colour between historic and contemporary specimens within each species and (ii) the differences in colour between species for each time period. For each comparison, we used bootstrapped, noise-corrected chromatic colour distances in units of just noticeable differences. If changes in colour for these comparisons are discernible, chromatic contrasts (ΔS) should be greater than 1. By contrast, if changes in colour are minimal, chromatic contrasts should be less than 1.

### 2.4. Statistical analyses

We conducted all analyses in R v. 4.0.2 [39]. The reflectance data were analysed and visualized by the R package ‘pavo’ v. 2.1.0 [38,40]. We smoothed all measurements (smoothing parameter 0.20) and produced average reflectance curves for historic and contemporary specimens of each oriole species. We obtained colorimetric variables and compared total brightness (B1), carotenoid chroma (S9) and hue (H3) in analyses detailed below. Brightness refers to the overall intensity, measured as the total photon flux [33], chroma is the purity of the dominant frequency, and hue is the dominant wavelength or frequency.

### 2.5. Fading hypothesis

To test the fading hypothesis, we compared orange and black colour variables (orange: total brightness, orange chroma, and hue; black: total brightness) in historic and contemporary specimens of both Bullock's and Baltimore Orioles from the hybrid zone. Because these specimens were collected in two discrete time periods (historic: 1956–1957; contemporary: 2016–2018), we used *t*-tests and Mann–Whitney tests to evaluate possible differences in colour variables between historic and contemporary specimens of each species separately. We checked that colour measures had a normal distribution using Shapiro–Wilks tests, and equal variances between historic and contemporary colour comparisons using *F*-tests. Two colour variables had unequal variances between historic and contemporary orioles: hue (H3) for Bullock's Orioles (*F* = 2.49, *p* = 0.05), and carotenoid chroma (S9) for Baltimore Orioles (*F* = 5.71, *p* < 0.001). For these two colour variables we used Mann–Whitney *U*-tests, and for the remaining colour variables we used *t*-tests. Because we conducted multiple tests on related orange colour variables, we corrected for false discovery rates following Pike [41].

### 2.6. Changes in diet hypothesis

We tested if changes in diet on the moulting grounds of Bullock's Orioles might explain the changes in colour we observed by comparing orange colour variables through time between Bullock's Orioles from within the hybrid zone and Bullock's Orioles from outside the hybrid zone. To evaluate possible changes in colour from orioles outside of the hybrid zone, we used a sample of 40 individuals, largely from breeding areas in Washington, Oregon, California and Nevada. We created three different linear models, each with a unique orange-colour variable as the dependent variable (total brightness, carotenoid chroma, hue), but each model contained the independent variables of date, location (within or outside of the hybrid zone), and interaction between date and location. If changes in orange colour in Bullock's Orioles were driven by changes on the moulting grounds, orioles from within and outside of the hybrid zone should show similar changes in colour, resulting in no significant interaction term in the models.

Data for date of collection, the co-variate in these linear models, differed between orioles from inside and outside of the hybrid zone. The orioles from outside of the hybrid zone spanned dates from 1902 to 2016, with relatively even sampling through time. By contrast, orioles from within the hybrid zone were collected in two discrete time periods in the 1950s and 2016–2018, so much so, that in other analyses we treated these two sampling events as categorical variables (i.e. historic and contemporary). Because of these sampling artefacts, data for the predictor variable ‘year’ were unevenly distributed through time for Bullock's Orioles from within the hybrid zone. To account for this, we re-ran these analyses using a subset of specimens from outside of the hybrid zone that were collected after the 1950s, so that date ranges were similar between Bullock's Orioles from inside and outside of the hybrid zone.

We constructed linear models using the R package ‘lme4’ v. 1.1–23 [42] and ensured that our data adequately fit assumptions of linear models using the package ‘DHARMa’ v. 0.4.5 [43]. Plots of model residuals and tests of multicollinearity revealed no concern in these models. We reported parameter estimates *β* (±s.e.), *z*- and *p*-values of the full models.

## 3. Results

### 3.1. General summary

**Figure 2.**
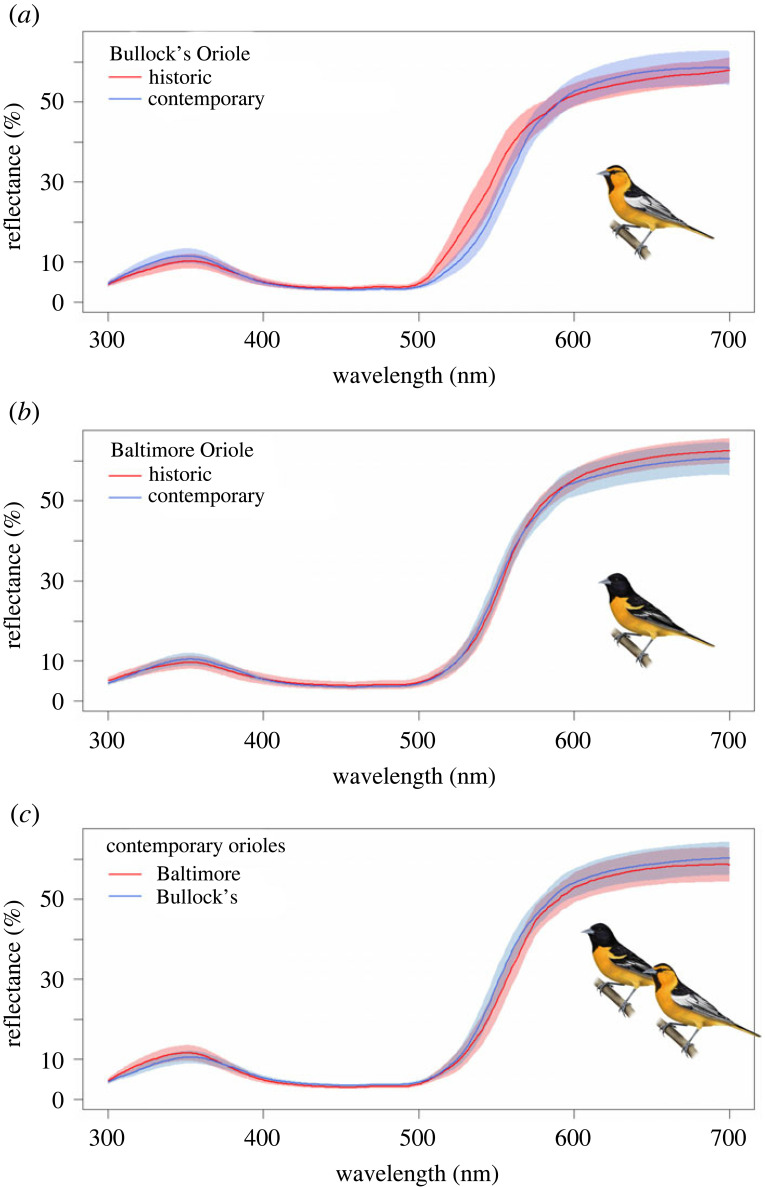
Bullock's Oriole plumage has shifted (*a*) to become more orange over time (note rightward shift in carotenoid-specific region of spectrum) whereas Baltimore Oriole plumage colour has not changed (*b*). The orange breast feathers of contemporary Bullock's Orioles have largely converged in colour to contemporary Baltimore Orioles (*c*). Reflectance spectra from plumage have been averaged for historical and contemporary specimens of both species, and shading around each averaged spectrum corresponds to the 95% CI.

**Figure 3.**
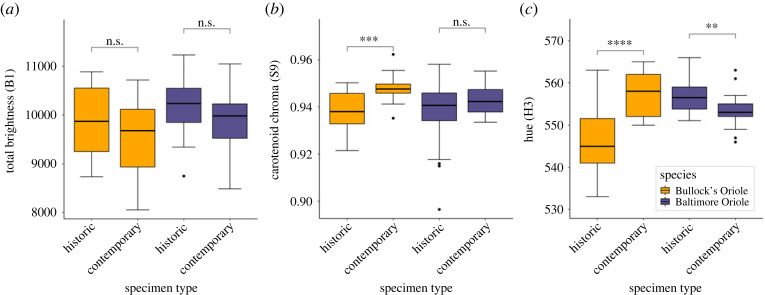
Fading does not explain the changes in orange colour between historic and contemporary Bullock's Orioles because similar patterns in orange colorimetric values are not observed in Baltimore Orioles. Panels show changes in three orange colour variables between historic and contemporary Bullock's Orioles (orange) and Baltimore Orioles (purple) for (*a*) total brightness, (*b*) carotenoid chroma, and (*c*) hue. Contrasts in colour values between oriole species are best observed by increases in carotenoid chroma in contemporary Bullock's, but not Baltimore Orioles (*b*), and increases in hue in contemporary Bullock's, but decreases in contemporary Baltimore Orioles. Significance codes are as follows: **** = *p* < 0.0001, *** = *p* < 0.001, ** = *p* < 0.01, n.s. = not significant.

In the roughly 60 years between sampling periods, the yellow-orange breast feathers of Bullock's Orioles have changed such that they now more closely resemble the orange breast feathers of Baltimore Orioles, the species with which they hybridize. These changes are clearly seen (i) when visually examining specimens (figure 1*a*), (ii) by the rightward shift in reflectance spectra for colour values that correspond to carotenoid pigments (approx. 500–600) (figure 2), and (iii) in colour metrics describing carotenoid-based plumages (figure 3). These changes in orange breast feathers of Bullock's Orioles show that contemporary Bullock's from within the hybrid zone have largely converged in orange colour to that observed in Baltimore Orioles (figure 2). By contrast, Baltimore Orioles show no shifts in orange colour variables that parallel those observed in Bullock's, indicating that this change in orange colour is only occurring in Bullock's Orioles.

### 3.2. Fading hypothesis

**Figure 4.**
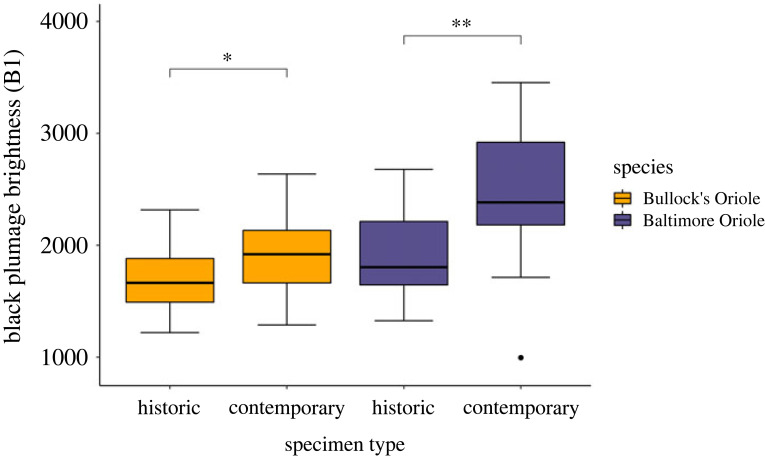
Changes in total brightness of the black throat patches in Bullock's and Baltimore Orioles show similar patterns of brighter feathers in contemporary compared to historic specimens as predicted by the fading hypothesis. Significance codes as follows: ** = *p* < 0.01, * = *p* < 0.05.

While some degree of plumage fading is likely to occur, this hypothesis does not explain the changes in orange colour variables observed in Bullock's Orioles. Measures of the black throat patches in Bullock's and Baltimore Orioles show the predicted pattern of duller black throats in historic specimens and, as predicted by the fading hypothesis, this pattern is repeated across both species (figure 4). However, this pattern is absent in orange colour variables, suggesting that the changes in orange colour observed in Bullock's Orioles are not driven by fading (figure 3). With the exception of the non-significant changes in total brightness, changes in orange colour variables are not repeated in consistent ways between Bullock's and Baltimore Orioles that share carotenoid profiles (figure 3; electronic supplementary material, table S3). The only significant change in orange colour observed between historic and contemporary Baltimore Orioles was hue, and this change was opposite to that observed in Bullock's Orioles. Thus, the lower values for hue in historic relative to contemporary Bullock's Orioles seem unlikely to be caused by fading because Baltimore Orioles show the opposite pattern where historic individuals have higher hue values relative to contemporary ones. This pattern is opposite to what would be predicted by the fading hypothesis (parallel shifts in colour in both oriole species between historic and contemporary samples), especially because these specimens have been stored in identical conditions for identical time periods. Taken together, these data offer little support for the fading hypothesis to explain the changes in orange colour we observed in Bullock's Orioles.

### 3.3. Changes in diet hypothesis

**Figure 5.**
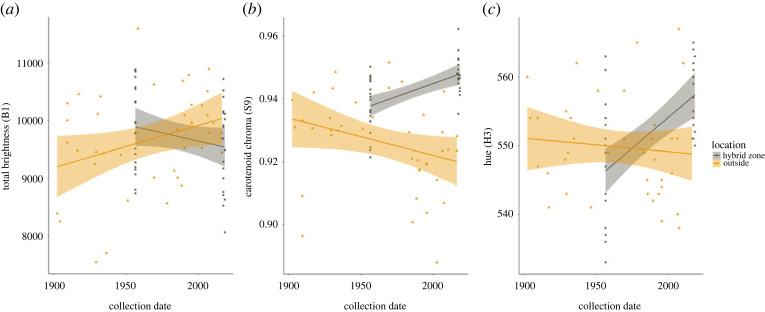
Changes in three colorimetric values through time in Bullock's Orioles from inside and outside of the hybrid zone. The significant interaction between date and location (inside versus outside the hybrid zone) across all three colour variables suggests that the changes in orange colour we observed in Bullock's Orioles are restricted to individuals within the hybrid zone and unlikely the result of changes in land use on the moulting grounds, which should have affected individuals from both inside and outside the hybrid zone in similar ways. Panels show contrasting changes in orange colour variables between Bullock's Orioles from inside (grey) and outside (orange) the hybrid zone for (*a*) total brightness, (*b*) carotenoid chroma and (*c*) hue.

Changes in diet or land use on the moulting grounds do not appear to explain the change in colour documented in Bullock's Orioles from the hybrid zone. Bullock's Orioles from outside of the hybrid zone show contrasting patterns through time in all three colour measures compared to Bullock's Orioles from inside the hybrid zone, as indicated by the significant interaction terms (figure 5, table 1). Colour profiles of Bullock's Orioles from inside and outside of the hybrid zone remain different, despite these birds moulting in a shared location, suggesting that whatever is responsible for the changes in colour observed within the hybrid zone is unique to that location. Re-running these analyses using a subset of specimens from outside of the hybrid zone that were collected after the 1950s revealed similar patterns. With the exception of brightness, all differences in colour variables in figure 5 remained significant (electronic supplementary material, table S1); for brightness, the trend from this subset of data was in the same direction as the original analysis in figure 5.

**Table 1.** Comparing changes in orange colour profiles of Bullock's Orioles from inside and outside the hybrid zone reveals contrasting patterns, as indicated by the significant interaction terms in all models, suggesting against the change in diet hypothesis. Summaries for linear models of three orange colour variables between Bullock's Orioles from inside and outside of the hybrid zone. Values indicate parameter estimates (*β*), standardized errors (s.e.), *z*- and *p*-values for total brightness, carotenoid chroma and hue. Bold values indicate significant values with *p* < 0.05.

parameters	*β*	s.e.	*z*	*p*
brightness				
intercept	9811.92	141.47	69.36	< **0.0001**
date	−0.012	0.01	−1.41	0.16
location (outside hybrid zone)	−114.50	188.34	−0.61	0.55
interaction (date*location)	0.04	0.01	2.50	**0.02**
chroma				
intercept	0.94	0.002	463.69	< **0.0001**
date	< 0.001	< 0.001	2.82	**0.006**
location (outside hybrid zone)	−0.014	0.003	−5.33	< **0.0001**
interaction (date*location)	< 0.001	< 0.001	−3.74	**0.0004**
hue				
intercept	548.8	1.25	438.21	< **0.0001**
date	< 0.001	< 0.001	4.97	< **0.0001**
location (outside hybrid zone)	0.95	1.67	0.57	0.571
interaction (date*location)	< 0.001	< 0.001	−4.27	< **0.0001**

### 3.4. Avian visual models

**Figure 6.**
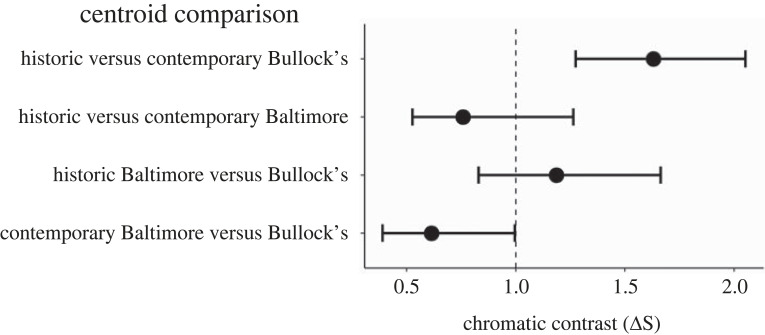
Historic and contemporary Bullock's Orioles show noticable shifts in orange colour, as indicated by chromatic contrasts (ΔS) greater than 1 in the top point. Orange colour profiles of contemporary Bullock's and Baltimore Orioles from the hybrid zone have largely converged in appearance, as indicated by the low ΔS value of the bottom point. Bootstrapped, noise-corrected chromatic colour distances in units of just noticeable differences show the degree of colour changes in Bullock's and Baltimore Orioles discernable by avian vision. Chromatic contrast values above 1.0 indicate discernibility.

Avian visual models revealed two important changes in orange colour of Bullock's Orioles from the hybrid zone that corroborate our observations of specimens. First, comparisons between historic and contemporary specimens of Bullock's Orioles show pronounced differences in chromatic contrasts (figure 6; chromatic contrast ΔS > 1.0), whereas comparisons of historic and contemporary specimens of Baltimore Orioles show largely similar chromatic profiles (ΔS < 1.0). These comparisons show that only orange colour profiles of Bullocks Orioles have changed through time. Second, comparisons of contemporary Bullock's and Baltimore Orioles from the hybrid zone have strikingly similar chromatic profiles, as indicated by the low ΔS value. By contrast, comparisons of historic Bullock's and Baltimore Orioles have noticeably different chromatic contrasts (ΔS > 1.0), consistent with observations of specimens (figure 1*a*). That the orange coloration of contemporary Bullock's Orioles has convergence in appearance to Baltimore Orioles suggests that these differences are perceived by birds and may be important for signalling to potential mates or competitors.

## 4. Discussion

Documenting changes in traits through time can be challenging because of uncertainty in which traits may change or because observed changes may be a consequence of long-term storage and lack biological meaning. Using a sample of Baltimore and Bullock's Orioles collected in 1955–1957 and 2016–2018, from the same locations along their hybrid zone transect, we documented an unexpected shift in the yellow-orange breast colour of Bullock's Orioles. Contemporary Bullock's Orioles have more orange breast feathers than historic Bullock's such that they now resemble Baltimore Orioles, the species with which they hybridize. This change in colour cannot be explained by fading because historic and contemporary specimens of Baltimore Orioles do not show parallel changes in colour through time (figure 3), despite identical storage conditions and similar carotenoid profiles in their feathers [18]. Similarly, changes in diet on the moulting grounds of Bullock's Orioles do not appear to explain this shift in colour because Bullock's Orioles from outside of the hybrid zone, which are thought to moult in the same areas as Bullock's Orioles from inside the hybrid zone [20,21], do not show similar shifts in orange colour variables (figure 5). These comparisons suggest that whatever is driving the changes in colour profiles of Bullock's Orioles is restricted to individuals within the hybrid zone.

Two hypotheses could account for these localized changes in Bullock's Oriole coloration: selection acting on standing genetic variation or an influx of genes through hybridization with Baltimore Orioles. Both hypotheses could produce the shift in colour described in Bullock's Orioles either through competition with Baltimore Orioles or other species (if converging in phenotype awards benefits to Bullock's Orioles through mimicry), or by sexual selection driven by female choice that favours more orange males [5]. The hybridization hypothesis could provide either genes that enhance the expression of carotenoid pigments in males, or genes in females that favour more orange coloration in males. While we cannot distinguish between selection acting on standing genetic variation and introgression, or the mechanism of selection (sexual, competition or both), we draw from knowledge of carotenoid-based plumages and other observations to propose that female choice for more orange plumage and an influx of genetic variation through hybridization with Baltimore Orioles may be responsible for the changes in Bullock's Oriole plumage we describe.

The dominant source of selection favouring this shift in colour (sexual selection driven by female choice or competition with Baltimore Orioles or other species) has important implications for distinguishing between selection on standing genetic variation and introgression. We suggest that the changes in Bullock's Oriole plumage colour are most likely driven by female choice for two reasons. First, carotenoid-based plumage characters are widely thought to signal individual quality [44–46], and play a role in mate selection in both Baltimore and Bullock's Orioles [47,48]. During pair formation, adult males of both species often perform bow displays where they point their bills upward, showcase the colour of their breasts, fan their tails, then bow forward while fluttering their wings [49]. For Bullock's Orioles, plumage colour may be even more important when nesting trees are limited. In these situations, Bullock's Orioles will breed in loose colonies within a single tree [50] and females often nest outside the territory boundaries of their social mates [51], suggesting that females may rely less on male territory quality and more strongly on individual signals of quality, such as the carotenoid-based breast and belly feathers. Second, the notion that convergence in colour of carotenoid-based breast feathers between Bullock's and Baltimore Orioles may mediate aggressive social interactions with Baltimore Orioles (or other species) seems unlikely given that overall plumage patterns between oriole species remain strikingly different. These persistent differences in plumage patterns between oriole species contrast with previous mimicry studies where convergence in plumage is striking [3,6,52]. Baltimore Orioles have entirely black heads, little white on their wings, and contrasting orange and black tails, whereas Bullock's Orioles have much more orange on their heads, more white on their wings, and less contrasting tail patterns [50]. These different colour patterns suggest that species recognition is unchanged by the comparatively subtle changes in colour we report, and that these changes in colour may be ineffective at changing social interactions with Baltimore Orioles or other species because overall appearances remain so different. Given that condition-dependent carotenoid traits are commonly used in mate attraction, we think it more plausible that these changes in plumage characters of Bullock's males are driven by Bullock's females that choose brighter males, and not by competitive social interactions with Baltimore Orioles or other species. Consistent with this idea, Richardson & Burke [47] showed that female Bullock's Orioles preferentially sought bright adult males, not duller subadults, when seeking extra-pair copulations. Similarly, recent work on introgression of a carotenoid-based plumage patch in hybridizing subspecies of Red-backed Fairy Wrens (*Malurus melanocephalus*) has shown that males respond equally to mounts of phenotypically different males (orange- versus red-backed) [53], but that females prefer red-backed males when given the choice between red- or orange-backed individuals [26].

If our supposition is correct—that these changes in plumage characters are most strongly driven by female choice—then this has implications for the origin of genetic diversity allowing changes in Bullock's Oriole plumage colour. If female preference in Bullock's for brighter males acted on standing genetic variation, then we should expect to see parallel shifts in colour in Bullock's from outside of the hybrid zone. Similarly, if the benefits of this shift in Bullock's colour were gained through social interactions with conspecific males, we should again see brighter males outside of the hybrid zone. Our data did not reveal these patterns. Instead, changes in Bullock's Oriole colour appear restricted to within the hybrid zone, and the orange breast feather coloration has converged with Baltimore Orioles, the species with which they hybridize. Moreover, because the magnitude of the shift in orange colour of Bullock's Orioles now closely matches orange colour profiles of Baltimore Orioles, it further suggests Baltimore Orioles as the source of these more vibrant oranges observed in contemporary Bullock's Orioles. These observations suggest that the changes in colour observed in Bullock's may be best explained by hybridization with Baltimore Orioles infusing local Bullock's genomes with genes that facilitate the metabolism/expression of carotenoid pigments in feathers. Back-crossed individuals are common within this transect of the oriole hybrid zone [17] suggesting that introgression added novel genetic material that enhanced the expression of carotenoid colours in Bullock's and females facilitated the spread of these beneficial alleles through sexual selection, as in other systems [26,54]. Introgression may also explain changes in Baltimore Oriole colour though time. Both species show opposite changes in hue values (figure 3*c*) with enhanced hue in contemporary Bullock's and muted hue in contemporary Baltimore Orioles, suggesting reciprocal introgression in some aspects of oriole plumage colour within the hybrid zone.

While the above scenario seems plausible, why has this shift in colour appeared only recently given that estimates of contact and hybridization between Baltimore and Bullock's Orioles range from 175 to 6500 years [16]? Two related hypotheses may provide some context for the relatively recent changes in colour we observed. First, combining genomes through hybridization is a gamble. Recombination risks breaking co-adapted gene complexes and thus stifling the spread of potentially beneficial alleles until, by chance, suitable gene combinations arise [55]. Recent work by Walsh *et al*. (unpublished) suggests the genomic basis of plumage coloration in Bullock's and Baltimore Orioles is regulated by many genes distributed across the genome, unlike other systems involving only a few genes [56,57]. This complex genomic architecture of colour suggests that compatible genetic combinations in hybrids or backcrossed individuals may be rarely acquired. Thus, one hypothesis is that those chance combinations have only recently arisen. Second, the habitat along the Platte River has changed with human activity [58], and these changes have likely promoted hybridization. The Platte River has long been used for irrigation and this has complex interactions with water levels, flood surges, and the extent of mature riparian forest along the riverbanks. Increased control over Platte River flows has promoted a more consistent river channel. As a result, riparian edges along the river have matured providing more suitable habitat for orioles, which likely facilitated increased hybridization and the westward spread of Baltimore Orioles [17].

Documenting genes associated with the expression of carotenoid pigments in vertebrates has been challenging because these pigments are, for the most part, acquired through diet and not produced endogenously. Moreover, the genetic basis of carotenoid pigmentation is complex as a suite of genes are thought to be associated with metabolic processing, transport, and deposition of carotenoids to specific traits [59,60]. Several recent studies using captive breeding experiments [57,61], across species comparisons [62], natural hybrid zones [63–65], and within-population correlations [66] of genotype–phenotype have established strong links between red plumage traits and the *CYP2J19* gene, a suspected ketolase gene thought to convert yellow dietary carotenoids into red ketocarotenoids [57,61]. Notably, hybrid zones between Red- and Yellow-fronted Tinkerbirds (*Pogoniulus pusillus and P. chrysoconus*) in South Africa [64] and between Red- and Yellow-shafted Flickers (*Colaptes auratus*) in North America [65] both revealed strong associations between the *CYP2J19* gene and yellow and red coloration across parentals and the variation in hybrid phenotypes. While Baltimore Orioles have about 29% more total carotenoids in their feathers than Bullock's Orioles from outside the hybrid zone, they have nearly twice the amount of ketocarotenoids (red pigments metabolically derived from dietary yellow carotenoids) [18], suggesting that Baltimore and Bullock's Orioles differ in their ability to convert dietary yellow carotenoids into ketocarotenoids. Variation in ketocarotenoid concentrations correlate with variation in hue across individuals in House Finches (*Haemorhous mexicanus*) [67]; recall that measures of hue between historic and contemporary Bullock's Orioles were the most strikingly different colour variable we described (figure 3). Taken together, these studies, especially those using avian hybrid zones to link the introgression of genes associated with carotenoid pigments and phenotypes [64,65], lend support to our supposition that hybridization with Baltimore Orioles may be responsible for the change in colour of Bullock's Orioles.

The shifts in colour we report inspire several additional questions. Baltimore and Bullock's Orioles hybridize along several river systems that traverse the Great Plains, USA. Examining these additional transects for repeated patterns of colour change would provide insight into how localized these shifts in Bullock's colour may be and if these shifts in colour may spread more broadly outside of the hybrid zone. Comparing the carotenoid composition of feathers between historic and contemporary orioles from the hybrid zone would provide a richer picture of the convergence in orange colour and if it is mirrored by a convergence in carotenoid composition between contemporary Bullock's and Baltimore Orioles. Finally, field studies that interrogate the ecological and evolutionary consequences of these shifts in plumage colour will provide a better understanding of the fitness consequences and dominant selective mechanisms (female choice or competitive social interactions) of this change in plumage colour.

Hybridization has long been recognized to play a creative role in evolution, as the influx of novel genetic material can allow for rapid trait evolution [8,68,69]. Comparing Baltimore and Bullock's Oriole specimens collected from the Platte River hybrid zone transect shows that contemporary Bullock's Orioles have converged in orange colour to the species with which they hybridize, the Baltimore Oriole. Bullock's Orioles now have noticeably more vibrant orange breasts and bellies but these phenotypic changes are observed only in individuals from the hybrid zone. The well-established role of carotenoid pigments in mate attraction and sexual selection suggests that the change in colour we describe in Bullock's Orioles may be driven by female choice acting on genetic material gained through introgression with Baltimore Orioles, which enhanced the expression of carotenoid pigments in Bullock's feathers. These changes in plumage coloration likely influence ecological and evolutionary dynamics of Bullock's Orioles, and probing these ideas awaits further study. Without the original specimens collected in the 1950s [29], these unforeseen changes in plumage colour likely would have gone unnoticed, illustrating how natural history collections deepen our understanding of microevolutionary changes in populations.

## Data Availability

Data and relevant code for this research work are stored in Github at https://github.com/younghasuh/Baltimore-Bullock-s-hybrid-zone and have been archived within the Zenodo repository: https://doi.org/10.5281/zenodo.7331680 [70]. The data are provided in electronic supplementary material [71].
